# Influence of Talocrural Joint Position on the Quadriceps Femoris Muscle Torque Measured with an Isokinetic and EasyForce^®^ Dynamometer in Young Adults

**DOI:** 10.3390/jfmk10030245

**Published:** 2025-06-27

**Authors:** Ivana Sović, Matija Brentin, Mirela Vučković, Andrica Lekić, Gordana Starčević-Klasan, Bojan Miletić, Hrvoje Vlahović

**Affiliations:** 1 Department of Basic Medical Sciences, Faculty of Health Studies, University of Rijeka, Viktora Cara Emina 5, 51000 Rijeka, Croatia; ivana.sovic@fzsri.uniri.hr (I.S.); gordanask@fzsri.uniri.hr (G.S.-K.); 2 Special Hospital for Medical Rehabilitation of Heart, Lung and Rheumatic Diseases-Thalassotherapia Opatija, Ul. Maršala Tita 188, 51410 Opatija, Croatia; 3 Department of Physiotherapy, Faculty of Health Studies, University of Rijeka, Viktora Cara Emina 5, 51000 Rijeka, Croatia; 4 Department of Clinical Medical Sciences I, Faculty of Health Studies, University of Rijeka, Viktora Cara Emina 5, 51000 Rijeka, Croatia

**Keywords:** EasyForce^®^ dynamometer, isokinetics, quadriceps femoris muscle, talocrural joint, torque

## Abstract

**Background**: Motor irradiation is a concept in which the muscles of the talocrural joint can influence the torque of the quadriceps femoris muscle. The aims of this study are to compare the effects of three different talocrural joint positions on the torque of the quadriceps femoris muscle and to analyze the differences in torque measurements between two dynamometers. **Methods**: 33 students from the Faculty of Health Studies at the University of Rijeka participated in the study. The torque of the quadriceps femoris muscle was measured using the Cybex CSMi isokinetic dynamometer and the EasyForce^®^ hand-held fixed dynamometer. The measurements were performed three times continuously for each talocrural joint position. **Results**: When measured with the isokinetic dynamometer (*p* = 0.210) and the EasyForce^®^ dynamometer (*p* = 0.925), no significant difference was found in the torque of the quadriceps femoris muscle between the three talocrural joint positions. The dynamometers measured the torque consistently and showed a significant, from very good to excellent correlation of the data (*p* < 0.001), but did not provide identical results due to constant and proportional differences. **Conclusions**: There was no significant influence of the three talocrural joint positions on the torque of the quadriceps femoris muscle under isometric contraction conditions without a specific angular velocity. The use of the two dynamometers led to similar but inconsistent results in the measurement of muscle torque. This inconsistency is not only because of the differences between the devices themselves, but also due to fundamental methodological differences in participant stabilization and testing procedures.

## 1. Introduction

The talocrural joint is characterized by biomechanical stability and the ability to adapt movement modulation in response to stimuli, which influences the functionality and coordination of the muscles. Mechanoreceptors achieve the stability of the joint and have a direct influence on the interaction of the surrounding musculature [1,2]. Muscle interaction is based on motor irradiation, in which the application of resistance to the musculature of one segment can facilitate the contraction of the musculature of another connected segment [3,4,5]. The concept of motor irradiation emphasizes the functional connectivity of muscle groups, such as the lower leg and thigh muscles, based on the equal sensitivity of spinal motor pathways and electrical impulses [6]. Understanding the concept of motor irradiation can help optimize the motor capabilities of different muscle groups, whether in movement or maintaining static positions.

Motor irradiation is related to the neurophysiological theory of motor responses of equal amplitude for the quadriceps femoris (QF) and tibialis anterior (TA) muscles in relation to the soleus muscle [7]. A deviation in the motor response was observed for the soleus muscle, as it belongs to the postural muscle group due to its slow-twitch muscle fibers [7]. The morphological similarities between QF and TA suggest a parallel activation pattern, which means that TA may indirectly influence the QF muscle strength and muscle torque [8]. Muscle torque is defined as the product of the muscle strength and the muscle moment arm [9]. The mechanical force generated by the TA around the knee joint results in greater muscle strength and isotonic QF muscle torque during active dorsiflexion (DF) of the talocrural joint [10]. Moreover, the DF position of the talocrural joint has a significantly greater effect on increasing muscle strength and isometric/isotonic torque of the QF muscle compared to the plantar flexion (PF) position and compared to the neutral position (N) of the talocrural joint [11,12,13]. Although there is an established theory of the interaction between QF and TA, there are considerations about the influence of PF position on the torque of the QF muscle [14]. To confirm the interaction between QF and TA, it is primarily necessary to investigate muscle strength capabilities and muscle torque using dynamometric methods.

The isokinetic dynamometer (IKD) is the gold standard for the biomechanical assessment of muscle capabilities in various pathological conditions of the musculoskeletal system in clinical settings and rehabilitation centers [15,16]. Hand-held dynamometry (HHD) offers a simpler and more cost-effective solution when IKD is not available for daily use. HHD dynamometers have already shown significant validity, sensitivity, and specificity in assessing the isometric torque of the QF muscle compared to the IKD [17,18,19]. It has been experimentally found that the IKD produces higher values of QF muscle torque compared to HHD, which is not due to differences in equipment but rather to the stabilization of the trunk [20].

The EasyForce^®^ dynamometer represents an innovation in the field of hand-held fixed dynamometry (HHFD) and has proven to be highly reliable for the assessment of thigh muscle strength due to its measuring range of over 1000 Newtons in comparison to some dynamometers in the HHD category, which have a measuring range of up to 700 N [21]. The data on the effect of talocrural joint position on different muscle groups of the thigh do not provide sufficient confirmation as to whether the results of muscle strength and torque are due to neurophysiological theories or the use of different methods and equipment.

Therefore, the purpose of this study is to compare the effects of the three different positions of the talocrural joint on the torque of the QF muscle measured with the IKD and the EasyForce^®^ dynamometer in students. The hypothesis is that the QF muscle torque is significantly higher in the DF position than in the PF and N positions of the talocrural joint and that there are no significant differences in the QF muscle torque measurements between the Cybex CSMi and the EasyForce^®^ dynamometer.

## 2. Materials and Methods

### 2.1. Participants

This study was conducted in 2024 at the University of Rijeka, Faculty of Health Studies, and at the Special Hospital for Medical Rehabilitation of Heart, Lung and Rheumatic Diseases—Thalassotherapia Opatija. This study involved 33 young students from the Faculty of Health Studies, of both genders, aged 18 to 20 years.

The inclusion criteria for participation in this study were a range of motion of more than 15° for the DF position and more than 20° for the PF position of the talocrural joint. Exclusion criteria were neurological diagnoses and traumatic injuries that could affect the normal range of motion and muscle ability.

The number of participants was selected based on the number of participants in previous studies, which ranged from 15 to 52 participants [10,11,12,13,14]. Participants signed an informed consent statement to participate in this study and also agreed to the use of anonymized photographs for publication of the study.

### 2.2. Testing Procedure

The devices used to measure the torque of the QF muscle were a Cybex CSMi isokinetic dynamometer (Computer Sports Medicine, Inc., NASA, Stoughton, MA, USA) and an EasyForce^®^ hand-held fixed dynamometer (Meloq AB, Stockholm, Sweden).

The IKD measures the torque in Newton-meters (Nm), and the obtained results are displayed graphically [22,23]. The characteristics of the HHFD are that it measures muscle strength in Newtons (N) and is a two-sided tensile dynamometer—one end has a hook connected to a fixed chain, while the other end has a hook attached to a cuff placed two centimeters (cm) above the lateral malleolus [24,25]. Before testing, participants’ range of motion was determined for DF and PF. The range of motion was tested using a standard goniometer. The neutral position of the talocrural joint is 90° and represents the angle formed by the foot relative to the lower leg. The range of motion in the talocrural joint for DF is 10–20°, and for PF, 40–55° [26]. The dominant leg was determined by having the participant stand in a relaxed, upright position while being pushed forward by the examiner. The leg that took the step forward was considered the dominant leg.

#### 2.2.1. IKD Testing Procedure

**Figure 1:**
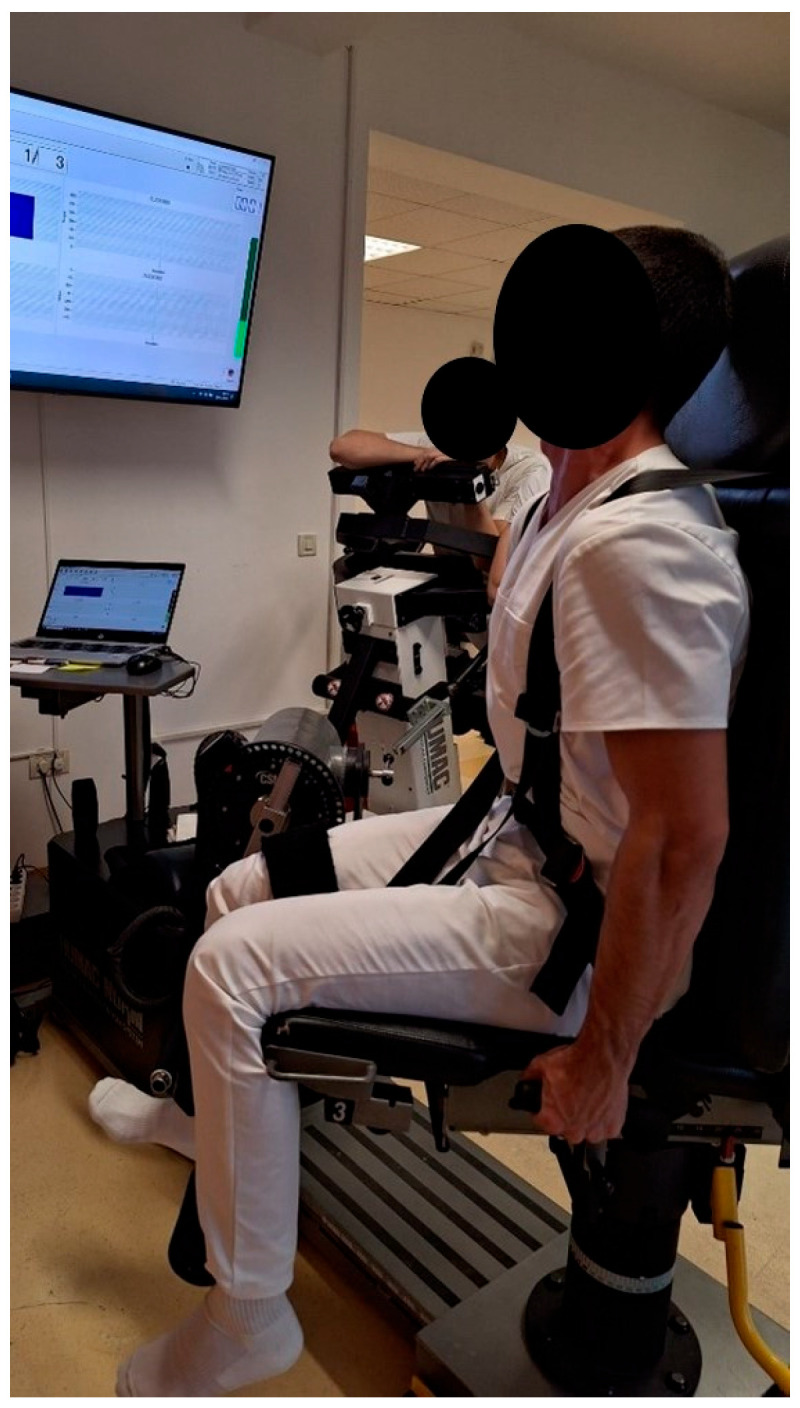
IKD test of the QF muscle torque with the DF position of the talocrural joint.

The QF muscle torque tests with the IKD dynamometer were performed in light clothing, without shoes, with arms at the sides, and in a seated position on the dynamometer chair with knees flexed at 90°. Maximum fixation was ensured by stabilizing the upper body at a 90° angle to the thighs. Participants actively performed DF at the talocrural joint and maintained a static position. The same procedure was used for the PF and N positions of the talocrural joint. After active positioning of the talocrural joint, participants attempted to extend the lower leg in the knee joint against resistance for five seconds (Figure 1). The results of the QF muscle torque measurements were expressed in Nm and measured continuously three times for each talocrural joint position. The final result of the QF muscle torque was the average of three measurements. QF muscle torque values were recorded using the HUMAC2015v15.000.0283:NORM version of the dynamometer computer program.

#### 2.2.2. HHFD Testing Procedure

**Figure 2:**
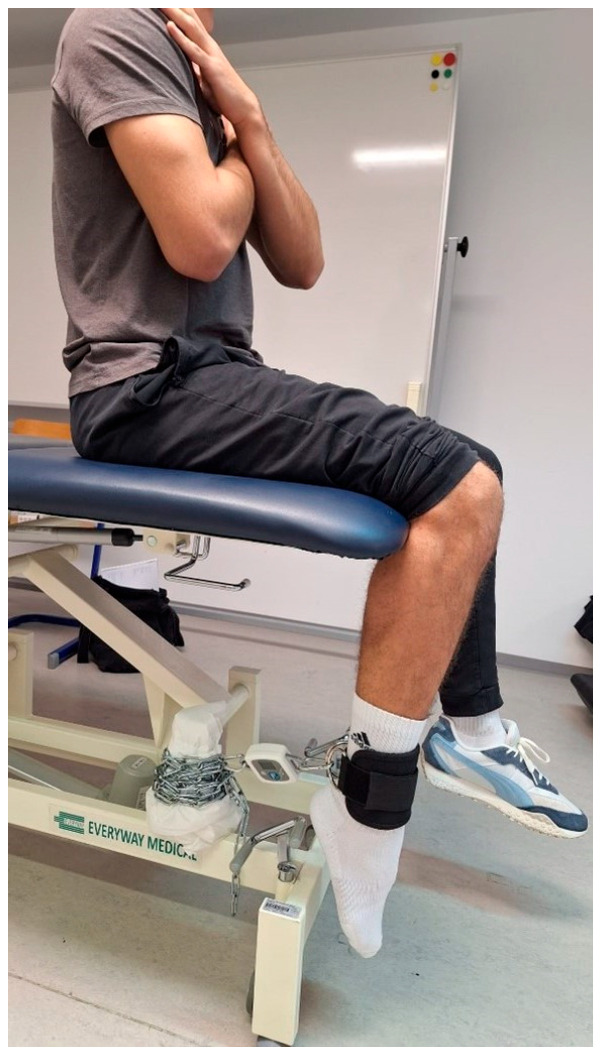
HHFD test of the QF muscle torque with PF position of the talocrural joint.

Testing the QF muscle torque with the EasyForce^®^ dynamometer also included measuring the muscle moment arm in meters (m) with a centimeter tape for each participant. The EasyForce^®^ dynamometer initially measures muscle strength in Newtons, which is not the same as torque in Newton-meters. The moment arm of the QF muscle represents the distance between the lateral condyle of the femur and a point five centimeters proximal to the distal end of the lateral malleolus [20,27]. One end of the dynamometer was attached to the table, while the other end was attached with a cuff to the area above the participants’ talocrural joint. The participant sat with hands crossed on the chest without back support and with knees in flexion at 90° (Figure 2).

The results of the QF muscle strength measurement were expressed in N and performed three times consecutively for each position of the talocrural joint. The final result of the QF muscle torque measurement was the mean of the three measurements.

The value of the muscle moment arm was converted from centimeters to meters and multiplied by the value of the QF muscle strength measured in N. The QF muscle torque measured with the EasyForce^®^ dynamometer was calculated using the following equation:

(1) $$\vec{M\;}=\;\vec{F}\;\text{×}\vec{\;d}$$

**Theorem** **1.**
$\vec{M}$ *stands for the QF muscle torque, expressed in Nm; $\vec{F}$ for the QF muscle strength, expressed in N; and $\vec{d}$ for the muscle moment arm, expressed in meters.*

By calculating the muscle torque in this way, it is possible to obtain values expressed in the same units of measurement, which allows for greater data variability and comparison between the Cybex CSMi and EasyForce^®^ dynamometer.

IKD test of QF muscle torque was performed on three consecutive days in January 2024, and the test with the EasyForce^®^ dynamometer took place in April 2024, also on three consecutive days. Due to the limited availability of devices, equipment, and test dates, strict randomization of the testing protocol was not possible. Instead, participants were offered available time slots and chose their test dates according to their availability. Although the protocol was not randomized, participants were evenly distributed across the test days, with eleven participants per day. It is important to note that the order of talocrural joint positions among the participants was not randomized. All participants followed the same protocol for testing QF muscle torque in the order of DF, N, and PF positions of the talocrural joint.

### 2.3. Statistical Analysis

The median, lower, and upper quartiles, and the range are given for all variables. The comparison of the effects of three different positions of the talocrural joint on QF muscle torque with IKD and HHFD was analyzed using the Kruskal–Wallis test and presented in a Box and Whisker plot diagram.

To evaluate the agreement of QF muscle torque results at different positions of the talocrural joint between the IKD and HHFD methods, Passing–Bablok regression with a Scatter plot diagram and Spearman’s correlation was used.

All data were analyzed using Statistica 14.1.0.8. (TIBCO Software Inc., San Ramon, CA, USA) and MedCalc 23.2.7. software (MedCalc Software Ltd., Ostend, Belgium). Statistical significance was assessed at a level of *p* < 0.05 with 95% confidence intervals (CI).

## 3. Results

### 3.1. Characteristics of the Participants

This study included 33 participants (*n* = 33). Of these participants, 21 were women (63.64%) and 12 were men (36.36%). Participants’ height, weight, and dominant leg data can be found in Table 1.

### 3.2. Comparison of the Effects of Three Different Talocrural Joint Positions on the QF Muscle Torque Measured with an IKD and HHFD

**Figure 3:**
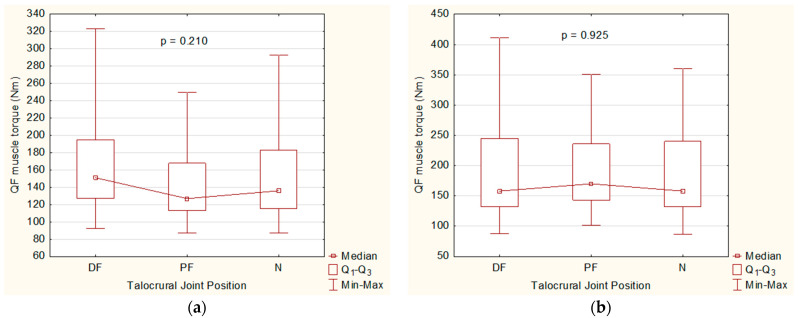
Comparison of the effects of three different talocrural joint positions on the QF muscle torque. (**a**) Box and Whisker plot diagram for the QF muscle torque variable measured with an IKD. (**b**) Box and Whisker plot diagram for the QF muscle torque variable measured with HHFD.

The median, as well as the lower and upper quartiles, of the torque of the QF muscle measured with the IKD were higher in the DF position, with 151.00 (127.00–195.00) Nm, than in the PF position, with 127.00 (113.00–168.00) Nm, and in the N position, with 136.00 (115.00–183.00) Nm (Figure 3a).

The median QF muscle torque measured with HHFD in the PF position, 170.00 (143.00–236.00) Nm, was higher than the same values of muscle torque in the DF position, 158.00 (132.00–245.00) Nm, and in the N position, 158.00 (132.00–240.00) Nm (Figure 3b).

There was no significant difference in QF muscle torque between the DF, PF, and N positions of the talocrural joint when measured with IKD (*p* = 0.210) and HHFD (*p* = 0.925) (Figure 3a,b).

### 3.3. Comparison of the QF Muscle Torque Values Between IKD and HHFD

The linear equation for the QF muscle torque variable measured with IKD and HHFD during the DF position is y = −37.77 + 1.42x. During the PF position, it is y = −40.58 + 1.62x, and during the N position of the talocrural joint, it is y = −36.64 + 1.49x (Table 2).

**Figure 4:**
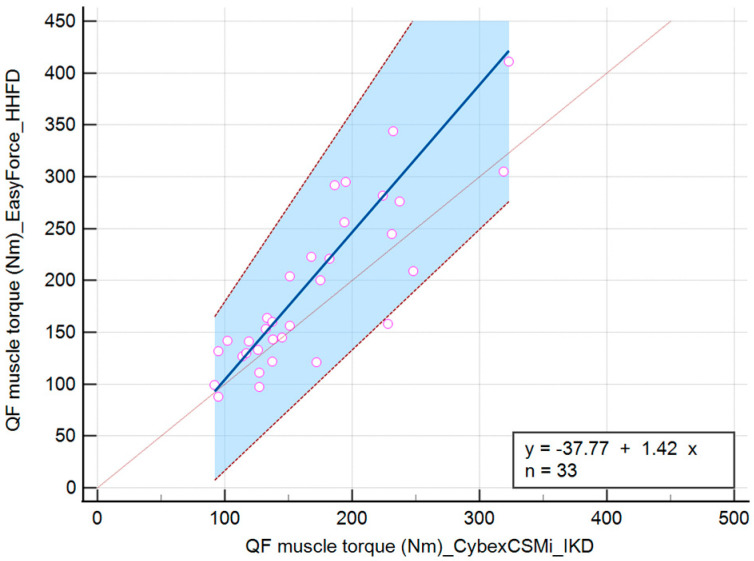
Scatter diagram comparing IKD and HHFD when measuring the QF muscle torque during the DF position of the talocrural joint.

QF muscle torque values during the DF position of the talocrural joint showed a significant difference in intercept (−37.77 (−99.67 to −3.57)) and slope (1.42 (1.16 to 1.83)) when comparing IKD and HHFD, indicating consistent and proportional differences between the methods (Figure 4).

**Figure 5:**
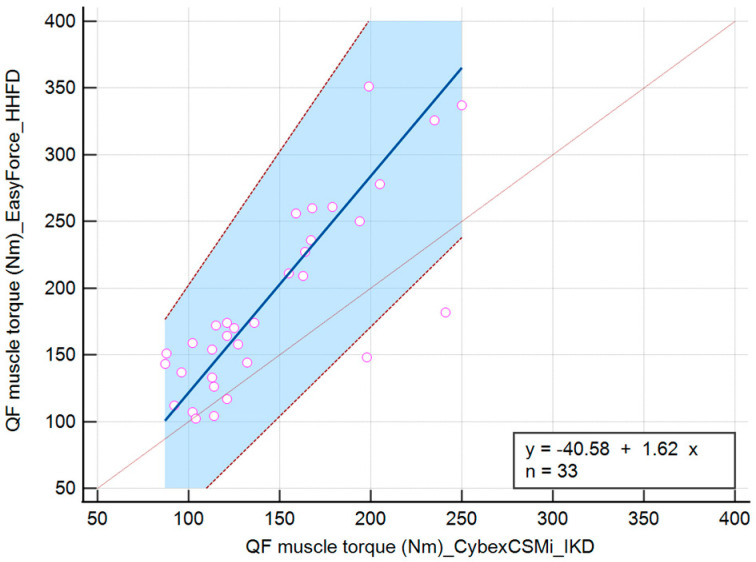
Scatter diagram comparing IKD and HHFD when measuring the QF muscle torque during the PF position of the talocrural joint.

The values of the torque of the QF muscle during the PF position of the talocrural joint showed no significant difference in the intercept (−40.58 (−97.00 to 2.50)). When comparing IKD and HHFD, there was a significant difference in slope (1.62 (1.34 to 2.00)), suggesting that there is not a constant difference but a proportional difference between the methods (Figure 5).

**Figure 6:**
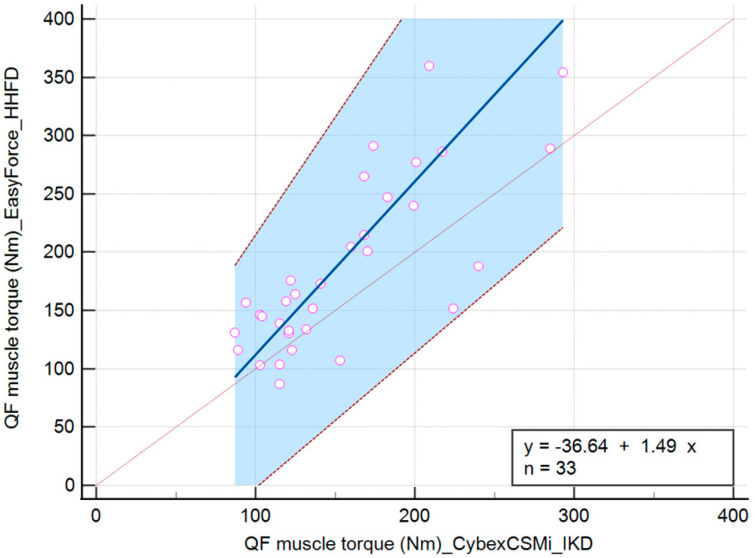
Scatter diagram comparing IKD and HHFD when measuring the QF muscle torque during the N position of the talocrural joint.

The values of QF muscle torque during the N position of the talocrural joint showed no significant difference in the intercept (−36.64 (−118.02 to 12.95)). When comparing IKD and HHFD, there was a significant difference in the slope (1.49 (1.16 to 2.02)), indicating that there is not a constant difference but a proportional difference between the methods (Figure 6).

### 3.4. Degree of Correlation Between the Measured Methods

The degree of correlation between the IKD and HHFD methods based on the QF muscle torque measurements in the DF, PF, and N positions of the talocrural joint was statistically significant (*p* < 0.001) and amounted to r(x,y) = 0.846 for DF, r(x,y) = 0.808 for PF, and r(x,y) = 0.773 for the N position (Table 3).

## 4. Discussion

The results of this study show that different positions of the talocrural joint have no significant effect on the torque of the QF muscle measured with IKD and HHFD. The results obtained could be due to the effect of neurophysiological mechanisms of muscle co-activation and constant and proportional differences in the measurement of QF muscle torque between IKD and HHFD.

### 4.1. Influence of Talocrural Joint Position on the QF Muscle Torque

Various muscle groups are involved in the generation and transmission of muscle force and torque in the kinetic chain. The interaction of the muscles is influenced by mechanical factors such as the joint position and the function of the muscle–tendon unit. At knee flexion angles greater than 90°, the length of the fascicles in the QF muscle increases and the pennation angle decreases, allowing maximal voluntary isometric contraction (MVIC) of the QF [28,29]. In our study, the torque and strength of the QF muscle were not compared at different knee joint angles, but the results may be related to the fact that the knee was positioned at 90°, which ensures efficient muscle contraction according to biomechanical principles. In addition to the position of the knee joint, the position of the talocrural joint also influences QF muscle strength and torque. Active DF of the talocrural joint has been shown to significantly increase knee extensor torque by 29% to 60% compared to PF and N positions, regardless of the type of contraction [10,11,13]. In addition, the DF of the talocrural joint acts as a stimulus and influences the intrafusal muscle fibers of the muscle spindle in TA, extensor hallucis longus (EHL), and extensor digitorum longus (EDL), so that the force generated by the contraction of TA, EHL, and EDL can influence the muscle strength and torque of the QF [30]. The results of our study cannot be compared with those of previous studies because the tests were not performed at isokinetic angular velocities in degrees per second (°/s) of 60°/s and 180°/s, and electromyography was not used to assess muscle activation. Previous studies have shown that fixed PF and DF positions of the talocrural joint have no significant effect on QF muscle torque or EMG activity when tested at angular velocities of 60°/s and 180°/s, respectively [31]. Higher values of QF muscle torque were observed at lower angular velocities, reflecting the influence of the neurophysiological mechanisms underlying static and dynamic muscle contractions [32]. The muscle tests in this study were performed under conditions of isometric contraction without a specific angular velocity, which allowed intermittent pauses in the activation of the motor neurons. These pauses facilitated the recovery of motor neurones and contributed to the maintenance of corticospinal excitability. To support the onset of motor irradiation, these pauses during intermittent isometric contractions reduced fatigue and preserved the efficiency of signal transmission from the higher brain centres through the corticospinal tract to skeletal muscle. In contrast, constant, prolonged dynamic contractions at different angular velocities (60°/s, 180°/s, and 300°/s) placed higher metabolic demands and accelerated muscle fatigue, which may compromise the integrity of the corticospinal tracts. As a result, corticospinal excitability decreases, and greater synaptic input to the motor cortex is required to maintain the desired level of muscle activation. If this increase is not possible or not sufficient to compensate for the reduced excitability, the ability of the central nervous system to recruit motor units is reduced. This leads to central fatigue and reduces the potential for motor irradiation. These mechanisms explain why neuromuscular control during prolonged dynamic contractions differs from that observed under intermittent isometric conditions [33,34]. In addition, the data collected in this study are not consistent with previous studies, as the higher median QF muscle torque was observed during isokinetic testing in the DF position, while the highest median QF muscle torque was observed during HHFD testing in the PF position. The highest median QF muscle torque in the PF position during the HHFD test can be explained by the biomechanical relationship between QF muscle torque and plantar flexor activation. It has been demonstrated that the plantar flexors in the closed kinetic chain pull the femur, tibia, and fibula posteriorly, facilitating the extension of the lower leg by the QF muscle [14]. These results cannot be related to those of our study because the torque of the QF muscle was tested in the open kinetic chain and not in the closed kinetic chain. Furthermore, in our study, the same median values of QF muscle torque were found for the DF and N positions in the HHFD measurements. This could be due to an uncontrolled displacement of the talocrural joint from the N position to the DF position as a compensatory kinematic adaptation to overcome resistance more easily. Overall, higher QF muscle torque values across different talocrural joint positions were recorded using the EasyForce^®^ dynamometer. However, in our study, full-body stabilization was not ensured during EasyForce^®^ measurements, which likely explains the higher QF muscle torque values compared to those obtained through isokinetic testing. In the seated position, the lack of thigh and trunk stabilization during EasyForce^®^ testing allowed for compensatory movements of the pelvis, trunk, and talocrural joint, which, according to the literature, can significantly influence the accuracy and outcomes of strength measurements [35]. To minimize compensation and ensure proper stabilization, the concept of force neutralization has been proposed in the literature. This involves stabilizing the proximal body segments to counteract the force generated by the knee extensors acting distally, and this neutralization capacity is further enhanced by trunk fixation [36]. The lack of complete stabilization led to an unconscious compensatory shift of the talocrural joint from the N to DF position, resulting in altered effective mechanics and muscle activation due to a kinematic adaptation facilitating easier overcoming of resistance. All these arguments can be confirmed by comparing the QF muscle torque measurements at different talocrural joint positions between IKD and HHFD.

### 4.2. Cybex CSMi vs. EasyForce^®^

IKD is an objective assessment of muscle capabilities that requires a trained examiner and expensive equipment. Clinical settings force the use of a simpler version of the IKD that is also economically viable. Economically viable versions are HHD dynamometers, a subset of which are HHFD dynamometers. IKD and HHFD dynamometers are used for scientific research purposes in extensive biomechanical studies. Given the complex characteristics of IKD dynamometers, the question arises whether HHD/HHFD dynamometers can compete as reliable and valid devices, and not only as economically viable versions. Previous studies have compared IKD dynamometers with different types of HHD dynamometers, such as the MicroFET2 (Hoggan Health Industries, Draper, UT, USA) and the Nicholas Manual Muscle Tester (Lafayette Instrument Company, Lafayette, IN, USA). The results showed that HHD dynamometers tend to display muscle strength values depending on the strength of the examiner applying the resistance [17,37]. A novelty in the field of dynamometry is the EasyForce^®^ dynamometer, which showed remarkable reliability in the assessment of muscle strength of the flexor and extensor muscles of the knee joint compared to the MicroFET2 dynamometer of the HHD category [24]. The results obtained enabled the development of a study to compare the EasyForce^®^ dynamometer with the IKD dynamometer in the measurement of muscle torque. In this study, the two dynamometric methods measure QF muscle torque consistently, as the data correlate from very well to excellent, but they do not provide identical results due to constant and proportional differences based on deviations in the regression analysis. The differences may be due to design and infrastructural differences between the devices, sensor activation, calibration, and measurement procedure. The lengthy preparation of the devices and the stabilization of the chain under limited infrastructural conditions represent an absolute limitation of the EasyForce^®^ Dynamometer for clinical practice. In this study, higher values of QF muscle torque were recorded with the EasyForce^®^ dynamometer compared to the IKD, which could be related to the insufficient stabilization of the subject and the manual calculation of QF muscle torque. Insufficient body stabilization during the HHFD tests and holding on to the handrails of the IKD dynamometer are possible consequences of the results obtained. It has been shown that higher values of knee extensor muscle strength were measured during the IKD test when the participants did not hold on to the handrails of the dynamometer but placed their hands on their chest [38]. Body fixation is necessary to avoid biasing the results, and it has been shown that higher muscle torque values were recorded with IKD compared to the HHD only because the participant is better stabilized [20]. In this study, the subjects had absolute body fixation during the IKD tests but held on to the handrails of the dynamometer. In the HHFD tests, the trunk was not fixed, but the hands rested on the chest. This suggests that energy conservation by the upper extremities, together with compensation by the trunk, may have influenced the recording of higher QF muscle torque values measured with the EasyForce^®^ dynamometer.

### 4.3. Recommendations for Clinical Use of the EasyForce^®^ Dynamometer

Before starting testing with the EasyForce^®^ dynamometer, one must ensure that the test environment addresses the infrastructural requirements for proper installation of the device. The test setup must be stable, with one end of the dynamometer attached with a hook to a chain that is attached to a fixed, immovable object, while the other end is attached to a cuff that is placed on the participant, in a location depending on which muscle group is being tested. A chain is a much more stable option than straps or elastic bands, as it reduces the likelihood of unwanted leg movement during the test. Any joint movement during the measurement can lead to a shift from isometric to isotonic contraction and, therefore, affect the validity of the data. Stabilization of the body during the test plays a crucial role in ensuring valid results. If the tests are performed in a seated position, participants should hold on to the edge of the table to prevent movement of the upper body. In the supine position, stabilization is naturally achieved through contact with the surface, although it is also possible to hold on to the edges of the table. Before measuring, it is important to ensure the correct positioning of the joints; for QF muscle strength testing, most commonly at 90° knee flexion, as even minimal deviations can affect muscle length and strength production. A small pillow or rolled-up towel can be placed under the knee to ensure an even and stable flexion angle. This prevents unwanted lowering or raising of the lower leg during the test, which could otherwise change the muscle length and influence the strength measurements. The maximum contraction should be maintained for three to five seconds. This duration of maximum contraction is considered in the literature [39,40,41], but individual adaptation is possible depending on the characteristics of the participants. We also recommend standardizing the verbal instructions and, if possible, providing visual feedback during the test to allow a uniform maximum effort. Finally, to improve reproducibility, it is advisable to document all test parameters, including body position, table height, chain length, and hook/chain contact points, to allow reliable replication in future studies. Taking these factors into account can significantly reduce measurement variability and improve the reliability of HHFD and EasyForce^®^ protocols. This supports their effective application in the clinical setting and improves consistency compared to isokinetic systems.

### 4.4. Limitations of This Study

The limitations of this study include the manual calculation of muscle torque based on the EasyForce^®^ dynamometer, which primarily measures muscle strength. To avoid subjectivity, several examiners must be consulted for the manual measurements. Additionally, a fundamental methodological inconsistency between test protocols was present, as full biomechanical stabilization of participants during EasyForce^®^ testing could not be ensured due to infrastructural limitations. This differs from isokinetic testing protocols like those used with the Cybex CSMi, which provide precise control of movement and stabilization. Future studies should address participant stabilization, especially of the upper extremity and trunk, when using the EasyForce^®^ dynamometer. Methodological limitations include the insufficient randomization of subjects during the IKD and HHFD measurements and the lack of randomization of the three talocrural positions between subjects. The consistency of the sequence of talocrural joint positions allowed standardization of the protocol, but is certainly recognized as a methodological limitation. Future studies should focus on the randomization of subjects according to the test protocol. Moreover, the EasyForce^®^ should be further tested for muscle strength and torque measurements across different muscle groups of both the upper and lower extremities, as well as the trunk.

## 5. Conclusions

The innovation of this cross-sectional study lies in the determination of the optimal position of the talocrural joint during the measurement of the torque of the QF muscle. Furthermore, this is the first evaluation of the use of the EasyForce^®^ dynamometer compared to the Cybex CSMi during the QF muscle torque test.

This study provided new insights into the theories of motor irradiation and showed that there was no significant influence of different positions of the talocrural joint on the torque of the QF muscle under isometric contraction conditions without a predetermined angular velocity. The use of two dynamometric methods led to similar but not identical results in the measurement of QF muscle torque, indicating the need for further optimization of the methodology.

Future research should focus on the position of the subjects and the use of a suitable dynamometer model to obtain more accurate and comparable results by testing different subjects and muscle groups.

## Figures and Tables

**Table 1:** Characteristics of the participants.

Variables	Me (Q_1_–Q_3_)	Min–Max	Title 1	Title 2	Title 3
Age (years)	19.00 (18.00–19.00)	18.00–20.00	entry 1	data	data
Gender	*n* (%)		entry 2	data	data ^1^
Female	21 (63.64)				
Male	12 (36.36)				
Height (cm)	172.00 (165.00–181.00)	155.00–198.00			
Weight (kg)	68.00 (62.00–80.00)	49.00–102.00			
Dominant leg	*n* (%)				
Right	31 (93.94)				
Left	2 (6.06)				

Legend: Me—median; *n*—sample size; Q_1_—lower quartile; Q_3_—upper quartile; Min—minimum; Max—maximum; cm—centimeter; kg—kilogram.

**Table 2:** Comparison of the two dynamometers.

Variables	Intercept (95% CI)	Slope (95% CI)	Linear Model Validity (*p*) *
DF (IKD/HHFD)	−37.77 (−99.67 to −3.57)	1.42 (1.16 to 1.83)	1
PF (IKD/HHFD)	−40.58 (−97.00 to 2.50)	1.62 (1.34 to 2.00)	0.93
N (IKD/HHFD)	−36.64 (−118.02 to 12.95)	1.49 (1.16 to 2.01)	0.30

Legend: DF—dorsiflexion; PF—plantar flexion; N—neutral position; IKD—isokinetic dynamometer; HHFD—hand-held fixed dynamometer; CI—confidence interval; *p*—probability value. * The Cusum test for linearity confirms the applicability of the Passing–Bablok method if *p* > 0.05.

**Table 3:** Degree of correlation between the IKD and HHFD.

Variables	Spearman R	*p*-Value	Strength of Correlation
DF (IKD/HHFD)	0.846	*p* < 0.001	Very good to very strong correlation
PF (IKD/HHFD)	0.808	*p* < 0.001	
N (IKD/HHFD)	0.773	*p* < 0.001	

Legend: DF—dorsiflexion; PF—plantar flexion; N—neutral position; IKD—isokinetic dynamometer; HHFD—hand-held fixed dynamometer; R—Spearman’s coefficient of rank correlation.

## Data Availability

The data generated by this research can be obtained from the corresponding author upon reasonable request.

## Abbreviations

The following abbreviations are used in this manuscript:

QF	Quadriceps femoris
TA	Tibialis anterior
DF	Dorsiflexion
PF	Plantar flexion
N	Neutral Position
IKD	Isokinetic Dynamometer
HHD	Hand-Held Dynamometry
HHFD	Hand-Held Fixed Dynamometry
Nm	Newton-meters
N	Newtons
m	meter
cm	centimeter
kg	kilogram
M	Muscle torque
F	Muscle strength
d	Muscle moment arm
Me	Median
*n*	Sample size
Q1	Lower quartile
Q3	Upper quartile
Min	Minimum
Max	Maximum
CI	Confidence Interval
*p*	Probability value
R	Spearman’s coefficient of rank correlation
EHL	Extensor hallucis longus
EDL	Extensor digitorum longus
MVIC	Maximal Voluntary Isometric Contraction
